# CFD simulation data of a pico-hydro turbine

**DOI:** 10.1016/j.dib.2020.106596

**Published:** 2020-11-28

**Authors:** Libia Cenith Alvear Pérez, Manuel José Anaya Acosta, Cristian Antonio Pedraza Yepes

**Affiliations:** Faculty of Engineering, Mechanical Engineering Program, Research Group CONFORMAT, Universidad del Atlántico, Puerto Colombia, Colombia

**Keywords:** CFD Simulation, Pico-hydro, Renewable energy, Hydraulic turbine

## Abstract

This paper presents the data obtained from the CFD simulation of a pico-hydraulic turbine designed for electric power generation in residential units. Pico-hydro turbine systems are a booming option that seeks to take advantage of small waterfalls and/or streams to generate electricity in a clean way. The data collection process was carried out through simulations developed to a model created in SolidWorks, with the help of the ANSYS CFX software in which the solver configuration was adjusted to the parameters suggested by the manufacturer's manuals (ANSYS) and the boundary conditions were determined based on the current regulatory standards for drinking water systems for Colombia. These boundary conditions influence the operation of the turbine, which is why three runs were carried out corresponding to three points of operation and to obtain the output pressure, the fall of the turbine and the hydraulic power that the device would utilise. The data, acquired in a strict manner, offers potential support for further research in the area and the possible manufacture of prototypes. It also encourages the development of simulations and laboratory tests of this type of device in which geometric factors or operating conditions could be modified.

## Specifications Table

**Table:** 

Subject	Mechanical Engineering.
Specific subject area	Computational modelling, hydraulic energy, renewable energy.
Type of data	Table Figure
How data were acquired	Computer aided design (CAD), Computational fluid dynamic simulations (CFD). Software: SolidWorks, ANSYS. Design and simulations carried out in a Workstation (manufacturer: Lenovo) with Intel Core i78850U processor, 16GB of RAM and NVidia Quadro P500 graphics.
Data format	Raw Analysed
Parameters for data collection	The CAD model created responds to several needs and design constraints to be adaptable to the drinking water network of a home (1/2" nominal diameter). Operating conditions and boundary conditions are given by pressure and mass flow, three operating points were established equivalent to each mass flow value. The generation of the mesh must comply with quality parameters such as Skewness and orthogonal quality. simulations must also achieve adequate convergence, as well as maintain imbalances of less than or equal to 1%.
Description of data collection	Firstly, the CAD model was created in SolidWorks, which contains the main geometric and dimensional characteristics of the device. The model was exported to ANSYS to generate the mesh and configure the solver. For each point of operation boundary conditions were established and several runs were executed in order to determine the rotation speed data. Once the rotation speed of the device was obtained, the final simulations were carried out in which the data on the pressure at the outlet of the turbine was obtained. In addition, the pressure drop through the device and the hydraulic power transferred by the water were calculated.
Data source location	Institution: Universidad del Atlántico Program: Mechanical Engineering City: Puerto Colombia Country: Colombia
Data accessibility	With the article

## Value of the Data

• The data contained in this article are important since they contribute to a research area of alternative methods of electricity generation with little development such as hydraulic pico-turbines at the residential level, providing data concerning the pressure drop and theoretical power in this device, allowing to know the performance of it. It also provides a geometric model with its dimensional data.
• The data obtained shown in this article provides useful information for research professionals in science and engineering areas, specifically in renewable energies with domestic applications. Likewise, the laboratories of fluid mechanics and hydraulic machines would take these data as a basis for the development of their investigations.
• The data obtained in this work can be used as basis for the application of fluid mechanics theory through CFD simulations with the aim of finding non-conventional devices (pico-hydraulic turbines) that generate electricity at a residential level, taking advantage of the flow energy in drinking water networks.
• The data presented are considerable relevance for research in the area of renewable energies, based on the geometric and dimensional characteristics presented in this work.
• This work is expected to encourage the development of devices that allow the generation of electricity from small water flows and drinking water systems, which are important for rural populations and/or those with problems in the supply of the electricity service.

## 1 Data Description

**Fig. 1:**
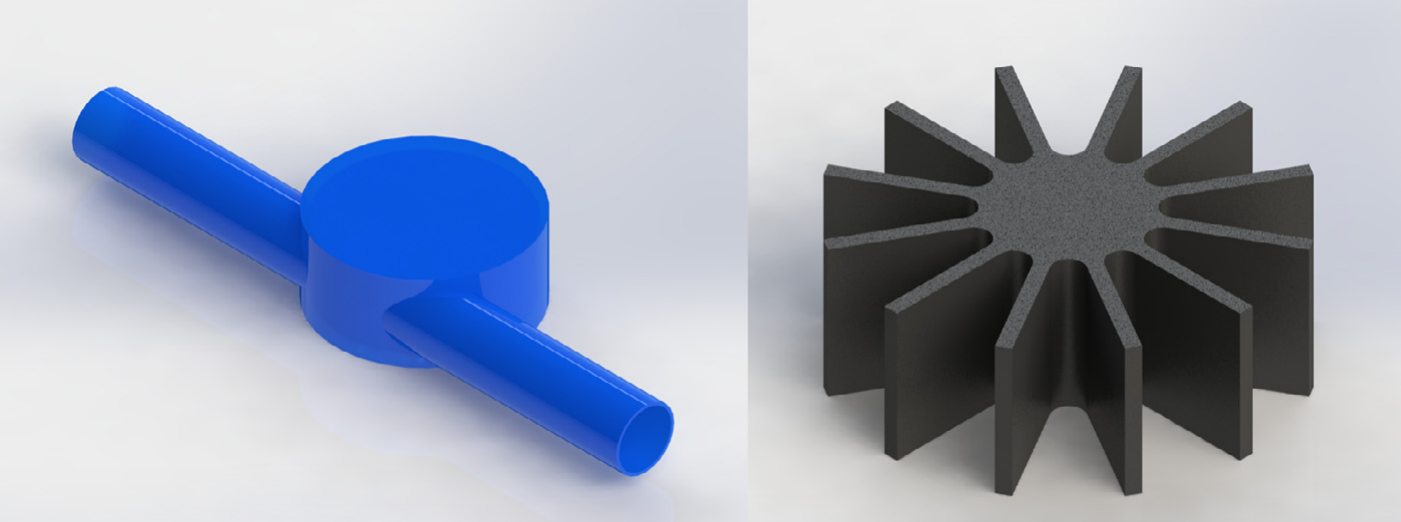
CAD model of pico-hydro turbine.

**Fig. 2:**
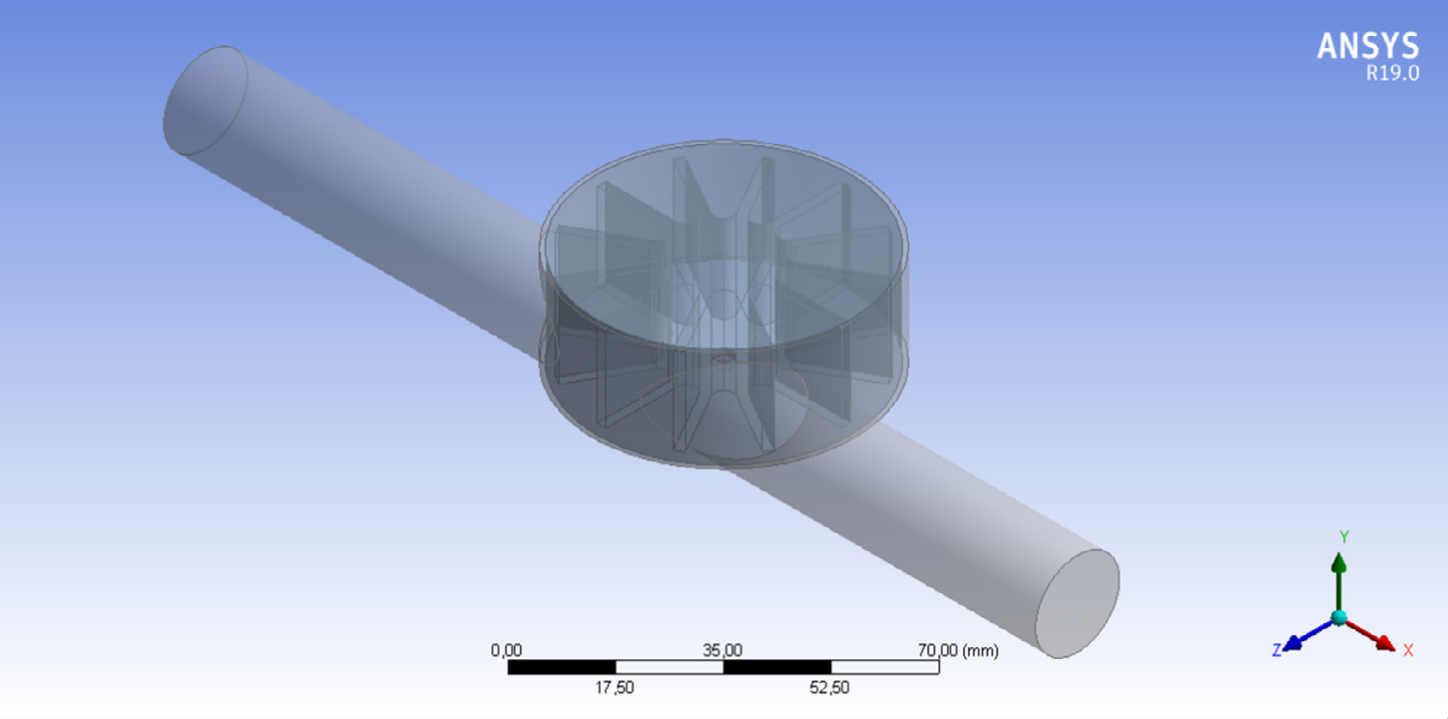
Control volume of pico-hydro turbine model.

**Fig. 3:**
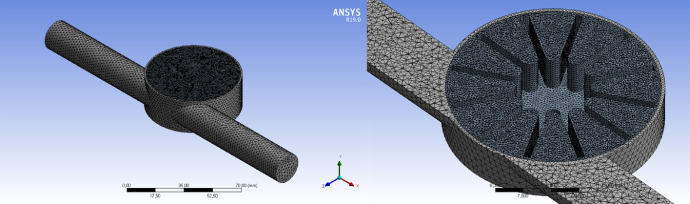
Mesh of model.

**Fig. 4:**
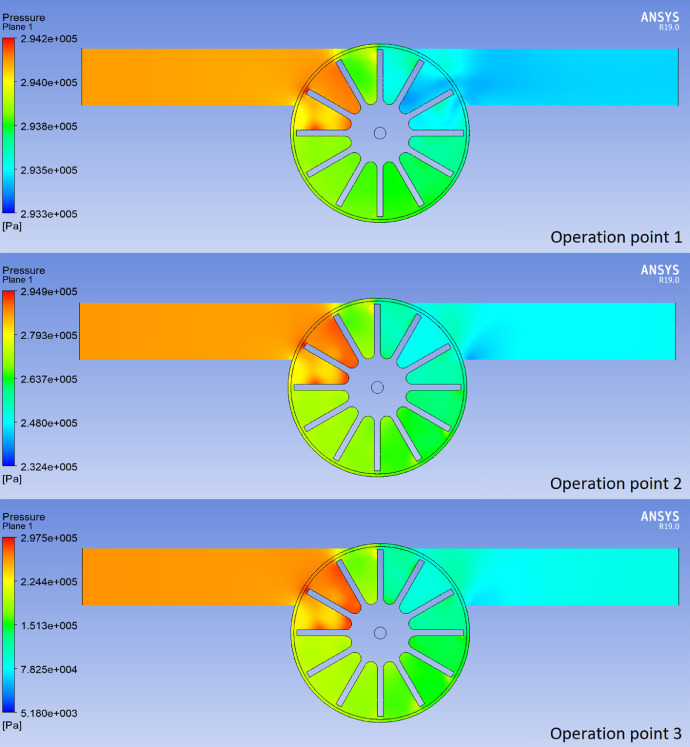
Pressure planes through the turbine.

**Fig. 5:**
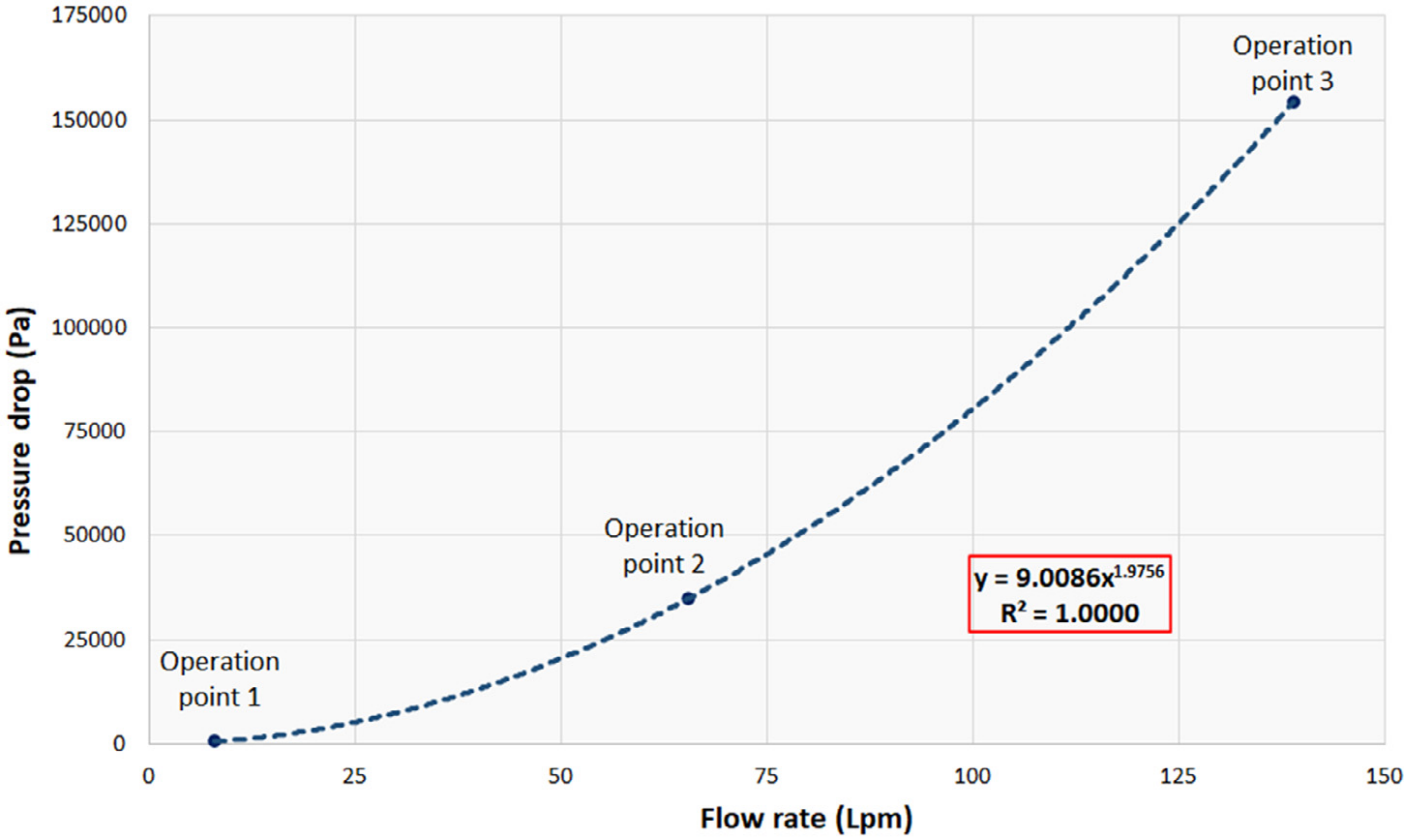
Pressure drop curve.

**Fig. 6:**
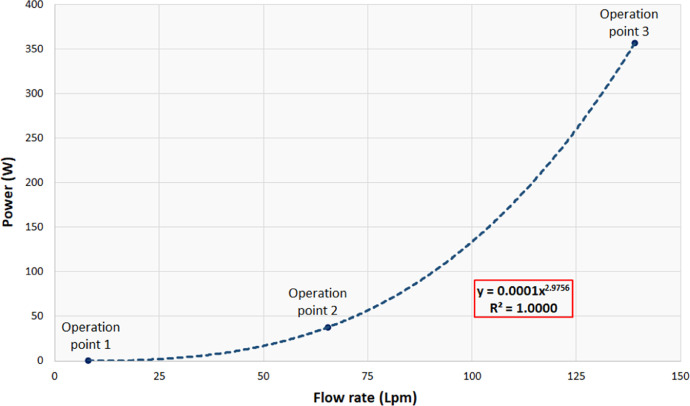
Hydraulic power curve.

Table 1 shows the corresponding measurements of the main parts that constitute the pico-hydraulic turbine model, established based on the coupling to a pipe of ½" nominal diameter. Fig. 1 shows the model created through SolidWorks, in which the rotor and the housing assembly are visualized; this CAD was created taking as reference the characteristics present in the rotor of a water meter. Table 2 shows the details of a mesh analysis in which 4 different sized mesh models were evaluated. Table 3 presents the design flow rate for the most commonly used hydro-sanitary devices in a common one-storey dwelling; the data was taken from the Colombian Code of Hydraulic and Sanitary Installations (NTC1500 third update of 16 August 2017) [1]. Table 4 shows the boundary conditions used in the simulations; pressure at the turbine inlet, obtained from the technical regulation for the drinking water and basic sanitation sector (Resolution 0330 8 June 2017) [2] and water mass flow based on the established flows. Fig. 2 shows the control volume of the pico-hydraulic turbine model exported to ANSYS. The geometric quality of the mesh is essential in a simulation, independently of the shape functions used [3]. Therefore, the mesh generated at the control volume of the pico-hydraulic turbine model is shown in Fig. 3. Previous runs were carried out in order to know the rotation speed of the turbine, in which the net torque is zero due to the zero angular acceleration. For this purpose, a parametric analysis was carried out with angular speed as the input parameter and net torque as the output parameter; the data corresponding to this step are shown in Table 5. The final simulations for each operating point were carried out once the rotation speed was known; Table 6 presents the fluid pressure values at the turbine outlet obtained in the simulation. Fig. 4 shows how the pressure changes through the turbine for each point of operation. From the data obtained in the previous step, the pressure difference and the energy theoretically absorbed by the turbine is calculated. Table 7 reveals the theoretical pressure and power drop calculated from the known data of pressure difference, flow rate and the specific weight of the water; the data corresponds to those generated at each operating point. Figs. 5 and 6 show the pressure drop and theoretical power at the three operating points respectively; furthermore, it can be seen from the graphs that the pressure drop is directly proportional to the theoretical power yielded by the water. From the raw data extracted from the simulations (Excel File), the necessary calculations were made to obtain the data shown in Tables 2, 5, 6 and 7. In addition, it was necessary to process the raw data for the generation of Figs. 5 and 6, corresponding to two variables of interest such as pressure drop and energy yielded by the fluid.

**Table 1:** Measurements of the pico-hydraulic turbine model.

Part	Measure (mm)
Input and output duct diameter	19.28
Height of rotor chamber	23
Rotor chamber diameter	60
Rotor height	20
Diameter swept by rotor	56
Thickness of rotor blades	2

**Table 2:** Analysis of different mesh models.

Characteristic	Mesh model 1	Mesh model 2	Mesh model 3	Mesh model 4
N° Elements	55571	416672	1007605	3300227
Stationary Size	5 mm	3 mm	2 mm	1.5 mm
Rotor Size	2 mm	1 mm	0.75 mm	0.5 mm
Skewness	0.2526	0.222	0.2177	0.2117
Orthogonal quality	0.745	0.776	0.7808	0.7867

**Table 3:** Theoretical flow rates for hydro-sanitary equipment.

Hydrosanitary equipment	Flow rate	
	L/min	m3/s
Dishwasher	10	1.666 × 10-4
Laundry room	15	2.5 × 10-4
Handwasher	8	1.333 × 10-4
Shower	11	1.833 × 10-4
toilet	95	1.583 × 10-3

**Table 4:** Boundary conditions required for simulations.

Operation point	Flow rate m3/s	Inlet pressure (Pa)	Mass flow kg/s
1 (minimum)	1.333 × 10-4	294191	0.13302
2 (medium)	1.092 × 10-3	294191	1.09
3 (maximum)	2.316 × 10-3	294191	2.311

**Table 5:** Parametric analysis of angular speed vs. net torque.

Design point	Operation point 1		Operation point 2		Operation point 3	
	Vangular (rad/s)	Net torque (Nm)	Vangular (rad/s)	Net torque (Nm)	Vangular (rad/s)	Net torque (Nm)
0	10	-0.0003589	55	0.0004639	100	0.0142394
1	6.9	8.1678E-07	55.65	2.7076E-05	122.3	0.0000202
2	6.95	-3.7497E-06	55.7	-4.5163E-06	122.35	-1.8735E-05
3	7	-7.9662E-06	55.75	-3.6099E-05	122.4	-5.8949E-05
4	8	-0.0001037	70	-0.00485023	145	-0.0187605

**Table 6:** Output pressure provided by the simulation.

Operation point	Output pressure (Pa)
1	293643
2	259334
3	139773

**Table 7:** Theoretical power calculated for each operation point.

Operation point	Pressure drop (Pa)	Theoretical power (W)
1	548	7.29E-02
2	34587	37.9979
3	154418	357.0127

## 2 Experimental Design, Materials and Methods

This work was developed in the facilities of the engineering laboratories of the Universidad del Atlántico. The model created in SolidWorks was simulated by applying computational fluid dynamics with the commercial code ANSYS CFX. The design and simulations were carried out on a Lenovo branded workstation, equipped with an Intel core i7 8550U processor, 16GB of RAM and NVidia Quadro P500 graphics card.

### 2.1 CAD modelling

The design of the model is based on the geometric characteristics of a single jet rotary vane water meter. The modelling started with the creation of the piece that represents the casing and the cover, taking into account that the turbine would be coupled to the pipe of a house (1/2" nominal) without reductions in the inlet and outlet to avoid pressure fluctuations due to the variation of the pipe diameter. Then, the 12 straight blade rotor was created (Fig. 1). It should be noted that the external geometry is not created in detail because it is unnecessary in this work. Table 1 shows the internal measurements used to create the model with the help of SolidWorks computer-aided design (CAD) software.

### 2.2 Simulation and data collecting

#### 2.2.1 Calculation mesh

Once the control volume was generated from the CAD in SolidWorks, this geometry was exported to ANSYS (Fig. 2) and afterwards the numerical discretization of the model was carried out through the generation of the mesh. The geometrical quality of the mesh is essential in a simulation independently of the shape functions used [3] and is checked through the skewness and orthogonal quality parameters, which vary in a range from 0 to 1, being better a value close to 0 in the skewness and a value close to 1 in the orthogonal quality.

To ensure the acquisition of appropriate results it is necessary to take several measurements regarding the number of elements, the size and the quality of them; therefore, four different mesh models were created and evaluated to determine which is the appropriate mesh for this research (Table 2). Models 1 and 2 are unsuitable due to the low number of elements and the wide variation between the parameters that guarantee the quality of the mesh; likewise, the difference of the skewness and the orthogonal quality is wide between models 2 and 3. Finally, in models 3 and 4 the variation of these parameters is very low compared to the increase in the number of cells generated, with model 4 having a high number of elements.

The calculation mesh chosen for the development of this work is number 3 because it is composed of 1007605 tetrahedral elements of size 2 and 0.75mm in the stationary and rotor domains respectively, a moderate amount with respect to the available computer resource (Fig. 3). In addition, the corresponding values for mesh quality are 0.2177 for Skewness and 0.7808 for orthogonal quality; thus ensuring excellent mesh quality [4].

#### 2.2.2 Boundary conditions

In ANSYS CFX, a valid configuration used to obtain a robust simulation is to set pressure at the inlet and mass flow at the control volume outlet [5]. In the turbine inlet section, an average value of those established in Articles 61 and 62 of Resolution 0330 of 8 June 2017 of the Ministry of Housing, City and Territory (Technical Regulation for the Drinking Water and Basic Sanitation Sector - RAS) was taken into account for the feed pressure; these articles provide the minimum and maximum service pressures in the distribution network [2], so it was decided to establish a pressure of 30 metres of water column equivalent to 294.191 kPa.

At the output of the control volume, the mass flow established as a boundary condition was calculated from the design flows of hydro-sanitary equipment shown in Table 3, based on standard NTC1500 third update of 16 August 2017 (Colombian Code of Hydraulic and Sanitary Installations) [1], of which the flows used were 1. 333E-4 m3/s, 1.092E-3 m3/s and 2.316E-3 m3/s; resulting in the following mass flow values: 0.1302kg/s, 1.09 kg/s and 2.311kg/s. The boundary conditions of the simulations are shown in Table 4.

#### 2.2.3 Simulation configuration

The study was carried out by dividing the operation of the turbine into three points of operation corresponding to the different mass flows determined above. This process was carried out due to the variability within the drinking water distribution network and consumption within the home; it also allows for a better understanding of the turbine's performance.

The k-ω turbulence model captures wall flows better and performs better in the presence of strong pressure gradients, however, after a series of test simulations, the k-ε model gave similar results to the k-ω model in a shorter time so it was decided to use the k-ε model. The convergence control of the simulation was set at steady state; the configuration of the convergence criteria was done, with residuals set at 1E-4 taking into account that it is a relatively low convergence, but may be sufficient for many engineering applications; it was necessary to provide sufficient relaxation to allow a convergent solution to be obtained [5]; as well as global imbalances with values below 1%.

#### 2.2.4 Obtained results

The development of the simulations was divided into two stages. In the first stage, the angular speed of the rotor was found. This was obtained by means of a parametric analysis, in which an arbitrary speed value was stipulated for each operating point (10 rad/s, 55 rad/s, 100 rad/s) and established as an input parameter. These values were modified until the speed corresponding to the net torque 0 (established as an output parameter) was found, that is, when the angular acceleration becomes 0, starting from the equation τ=Iα. Table 5 shows the values of angular velocity and net torque evaluated in the parametric analysis.

The next stage was to obtain the fluid pressure at the turbine outlet. This is obtained from the execution of the simulations once the rotation speed for each operating point was found. After several runs it became clear that, by varying the angular speed to the order of hundredths or thousandths of a unit (rad/s), the torque variation is very low, so it was decided to select the values shaded in Table 5 with a tolerance of five hundredths.

With the data corresponding to the outlet pressure (Table 6), the pressure drop through the turbine was calculated using the equation $\Delta p=p_{out}-p_{in}$. Then, the hydraulic power yielded by the water was calculated from the equation P=γQH; in which γ is the specific weight of the water, Q is the flow rate at the point of operation and H is the pressure difference expressed in meters. This data for each operating point is shown in Table 7. Fig. 4 shows a pressure plane corresponding to each operating point and Figs. 5 and 6 show the pressure drop and calculated power curves, respectively.

## Declaration of Competing Interest

The authors declare that they have no known competing financial interests or personal relationships which have or could be perceived to have influenced the work reported in this article.

## Appendix Supplementary materials

Image, application 1
